# The medium-term sustainability of organisational innovations in the national health service

**DOI:** 10.1186/1748-5908-6-19

**Published:** 2011-03-14

**Authors:** Graham P Martin, Graeme Currie, Rachael Finn, Ruth McDonald

**Affiliations:** 1 Department of Health Sciences, University of Leicester, Leicester, UK; 2 Business School, University of Warwick, Coventry, UK; 3 Management School, University of Sheffield, Sheffield, UK; 4 Business School, University of Nottingham, Nottingham, UK

## Abstract

**Background:**

There is a growing recognition of the importance of introducing new ways of working into the UK's National Health Service (NHS) and other health systems, in order to ensure that patient care is provided as effectively and efficiently as possible. Researchers have examined the challenges of introducing new ways of working--'organisational innovations'--into complex organisations such as the NHS, and this has given rise to a much better understanding of how this takes place--and why seemingly good ideas do not always result in changes in practice. However, there has been less research on the medium- and longer-term outcomes for organisational innovations and on the question of how new ways of working, introduced by frontline clinicians and managers, are sustained and become established in day-to-day practice. Clearly, this question of sustainability is crucial if the gains in patient care that derive from organisational innovations are to be maintained, rather than lost to what the NHS Institute has called the 'improvement-evaporation effect'.

**Methods:**

The study will involve research in four case-study sites around England, each of which was successful in sustaining its new model of service provision beyond an initial period of pilot funding for new genetics services provided by the Department of Health. Building on findings relating to the introduction and sustainability of these services already gained from an earlier study, the research will use qualitative methods--in-depth interviews, observation of key meetings, and analysis of relevant documents--to understand the longer-term challenges involved in each case and how these were surmounted. The research will provide lessons for those seeking to sustain their own organisational innovations in wide-ranging clinical areas and for those designing the systems and organisations that make up the NHS, to make them more receptive contexts for the sustainment of innovation.

**Discussion:**

Through comparison and contrast across four sites, each involving different organisational innovations, different forms of leadership, and different organisational contexts to contend with, the findings of the study will have wide relevance. The research will produce outputs that are useful for managers and clinicians responsible for organisational innovation, policy makers and senior managers, and academics.

## Background

There is a growing evidence base on the challenges of introducing new ways of working into complex organisational environments such as the UK's National Health Service (NHS). This evidence base covers the difficulties of achieving changes in professional bureaucracies infused with powerful institutional forces and the interventions that can be developed in order to increase the likelihood that such changes are accepted by the diverse stakeholder groups who will determine success or failure. However, there is considerably less knowledge of what happens after the initial 'push' for adoption of an organisational innovation of this kind has ended. In the short term, a new way of working may be developed, put into practice, and made to work, but what happens after the immediate campaign to introduce organisational change--for example, a policy mandate, a campaign to convince stakeholders of the worth of change, or short-term pump-priming money--ceases? This study will build on the existing literature on the uptake of new ways of working in the NHS, and on the emergent literature on the medium- and longer-term maintenance of these new ways of working, to produce new knowledge about what helps and hinders sustainability of such organisational innovations.

The existing literatures on change management, diffusion of organisational innovations, and public policy and management provide important lessons on the nature of the challenges relating to instituting, sustaining, and spreading change in the NHS and other complex public-service organisations. Recent literature in these fields has diverged from traditional models of the uptake and diffusion of innovations to be found in accounts such as that of Rogers [1]. Increasingly, this literature emphasises instead that 'the dissemination of innovations is not necessarily a linear process', but one in which 'rational, institutional and political forces' are implicated [2]. There is an increasing recognition of the importance of the complex nature of the public-service environment [3], as well as of the fact that organisational innovations are rarely so simple that they can be implemented without implications for wider practices, care pathways, and professional jurisdictions [4]. The implementation of such organisational innovations in public-service professional bureaucracies such as the NHS is thus a much more 'messy, dynamic, and fluid' [5] process than the linear 'S-curve' of innovation diffusion would suggest.

This has important implications for those seeking to introduce, replicate, and sustain change in the NHS. New ways of providing services will not translate simply into practice, even if backed by a substantial evidence base. Rather, they are likely to require considerable negotiation and political action. There is a growing evidence base on the kinds of interventions that can encourage uptake of organisational innovations, such as leadership distributed across the professional groups affected by the change [6-8], efforts to align innovations with wider group interests and policy pressures [9], and pursuing uptake as a process of *adaptation *to local need and context rather than simple *adoption *of a potentially inappropriate innovation [4]. Uptake is also more likely where certain contextual conditions are in place, such as strong interprofessional and interorganisational networks, and a receptive organisational culture [10,11]. Some aspects of Pettigrew *et al*.'s [12] model of a receptive context for organisational change might also be seen as applying to 'bottom-up' organisational innovations led from frontline clinicians and managers, with its identification of external pressures, skilled leadership, management-clinician relationships, supportive culture, clear policy/strategy, interorganisational networks, clear priorities, and fit between the change agenda and the organisation. These kinds of active interventions and contextual conditions are all the more crucial to the chances of change where organisational innovations emerge from the bottom up, led by individual clinicians or managers with 'good ideas' rather than driven by policy makers or by powerful organisations such as the National Institute for Health and Clinical Excellence (NICE) [8,13].

These factors are likely also to be important in work aimed at sustaining organisational innovations that have been successfully introduced. Some factors (*e.g*., a supportive organisational culture) are likely to come into play earlier on in the introduction of an organisational innovation, whereas others are likely to be more important in sustaining, maintaining, and routinising change (*e.g*., interorganisational relationships). However, there may also be further, divergent factors involved in ongoing sustainability of change. Over time, initial favourable conditions become less important, and the question becomes one of how far 'this innovation has the capacity to continue to adapt to current and foreseeable system conditions' [14]. To date, however, there has been little research on the question of the medium- and longer-term sustainability of organisational innovations. As Fitzgerald and Buchanan [15] note, 'in most studies of change, the focus has been with the "front end", with initiation, resistance, and implementation', with little attention paid to 'the process of change over a longer time frame'. In their systematic review of innovation in service organisations, Greenhalgh *et al*. [16] similarly found evidence to be 'very sparse', with a 'near absence of studies focusing primarily on the sustainability of complex service innovations'.

Thus there is a need for more research on how to mitigate the 'improvement-evaporation effect', as the NHS Institute [17] has termed it, and in particular, on the factors associated with successful sustainability and routinisation of organisational innovations [14,18]. In particular, what strategies--including but not limited to those outlined above--are required in establishing change that is robust enough to survive and thrive in a competitive NHS environment subject to changing priorities and finite resources, without the support of a top-down push by policy makers? This research seeks to provide answers to these questions by following four more or less bottom-up organisational innovations from a previous study carried out by the investigators. These innovations, each providing clinical-genetics services in a novel way that deviated from established practice in the field, were each initially successful in instituting new ways of working and obtaining follow-up funding after initial pilot money ceased. Having tracked them during the process of establishing their innovative ways of working and sustaining these in the short term through local funding in a previous evaluation, this research follows them through their medium-term efforts at consolidating change and ensuring their ongoing viability.

### Research question

The principal research question that the study seeks to address is the following: What helps and hinders the medium-term sustainability of micro- and meso-level organisational innovations in the NHS?

### Aims and objectives

The aims and objectives of the study are as follows:

• To carry out qualitative, comparative case-study research at four sites in which a novel way of delivering genetics services has been sustained in the period following pilot funding from the Department of Health and to combine this with secondary analysis of data previously collected in these sites as part of an evaluation of genetics service initiatives.

• To use this work to develop theoretically informed, generalisable knowledge about the facilitators and barriers in the sustaining and establishment of innovative approaches to service delivery and organisation in the medium-term period following initial introduction. As well as contributing to the academic evidence base, these lessons will be of use to NHS policy makers, managers, and clinicians involved in creating receptive contexts and acting effectively to support the ongoing survival and development of novel ways of delivering services, beyond initial funding decisions.

• To disseminate these findings through various means, including via National Institute for Health Research (NIHR) Collaborations for Leadership in Applied Health Research and Care (CLAHRCs) to reach researchers and practitioners involved in the translation of new ways of working into routine NHS practice, via partnerships with Macmillan Cancer Support and the NHS Genetics Education and Development Centre to reach practitioners involved in developing new services in these fields, and through peer-reviewed publications targeting the academic community.

## Methods/design

This study consists of a follow-up study that builds on a recently completed (autumn 2008) evaluation of new approaches to providing genetics services in the NHS. The original evaluation was a qualitative, longitudinal study that examined 11 theoretically sampled cases of organisational innovation in the provision of genetics services, involving, variously, reconfigured care pathways; alternative settings of care across the primary, secondary, and tertiary sectors; and new divisions of responsibility between professions and specialities. This study involves further research in a subsample of 4 of the 11 sites, all of which were initially successful in sustaining their work beyond their pilot periods but which differ in their clinical focus, health-service sector, and interprofessional division of labour. By conducting secondary analysis of the original data set and then revisiting these sites around 30 months after the initial three years of fieldwork were completed, this comparatively small-scale study will create a rich, longitudinal data set that allows a nuanced understanding of the medium-term sustainability of these services, taking account of contextual and process differences between the theoretically sampled sites [19] and understanding contemporary challenges and resolutions in their historical, path-dependent contexts [20].

### Design and theoretical/conceptual framework

The research is informed by the empirical and theoretical literature outlined above. While building on traditional notions of innovation adoption, diffusion, and sustainability, recent research has also drawn attention to the deficiencies of linear models of uptake in relation to complex public-service organisations and professional bureaucracies such as the NHS [4,6,10,16]. Instead, these studies emphasise the need to account for complications in the uptake and sustainment of organisational innovations by viewing these as processes of negotiation among multiple interested stakeholder groups [4] and by understanding sustainability in the contexts of organisation, system, and history [12]. This requires a simultaneous attention to both structure and agency, acknowledging the powerful institutions that structure organisational practices, professional relationships, and individual actions but also recognising the ability of individuals and groups to challenge and transform existing institutions [21]. Understanding the processes through which institutions are transformed requires close attention to particular settings to provide insight into how actors embedded in particular fields seek to implement and sustain change [22].

In keeping with these conceptual frameworks, the study deploys a theoretical sampling strategy to select four sites from the prior study that converge and differ in respects that (based on the literature and on the contextual understanding developed in the earlier evaluation) are likely to determine the challenges around sustainability and appropriate responses to these challenges (see 'Sampling, setting, and context' below), giving the research wider relevance across the health service and aiding generalisability [19]. The study aims to understand the challenges faced in sustaining organisational innovation beyond the initial stages of adoption and adaptation, which have formed the focus of most prior research [15,16], and how various factors relating to (interalia) the organisational structures of different health-service contexts, the characteristics of the organisational innovation being sustained, and the agency of various influential stakeholders interact to affect the prospects for the sustainability of the innovation. The study will pay particular attention to the movement from initial sustainability with local money to the medium-term process of 'embedding' these ways of delivering services in the fabric of the local NHS. As noted above, little research has addressed this question up until now, with most inquiry focused on the front end of service innovation. However, the emergent literature [23]--as well as our previous evaluation and some of the findings it has produced [8,24]--indicates some of the issues worthy of particular attention. Sibthorpe *et al*. [14], for example, suggest that while favourable conditions (*e.g*., a risk-accepting organisational environment) may be crucial in enabling an innovation to get off the ground, these become less important over time as service moves into sustaining initial gains, and so the ability of a service to demonstrate its effectiveness and worth becomes more important--as too does the skill of leaders and teams in generating the maximum political capital from this. Our own research from the earlier evaluation--which covered not just the establishment of the organisational innovations but also their initial efforts, successful and unsuccessful, in making these sustainable--affirms this suggestion to some extent, highlighting the importance of effective, dispersed leadership in ensuring that a critical mass of powerful actors in the local network of organisations is aware of the advantages of the new model of service delivery [8]. However, our findings also indicated that the process may be more cyclical, with the achievement of sustainability requiring ongoing innovation and reinvention to appeal to the divergent criteria used to judge success by different audiences (referring clinicians, general managers, primary care commissioners), at least in the short term [24]. In some of our cases, initial sustainability was achieved through the mobilisation of more or less informal coalitions of clinicians, managers, and service users in support of ongoing funding; others pursued a strategy of alignment with formal organisational priorities to secure the buy-in of senior-level managers and prevent improvement evaporation [8,9,25,26]. As described in more detail below, this new study will enable us to revisit these findings--and the way in which different organisational contexts demand different strategies, with varying levels of success--specifically in the light of the emergent literature on sustainability and to consider them explicitly in addressing the transition from introduction, through to initial sustainability, through to local funding, to the medium- and longer-term sustainability that secures the place of services as established components of the local health economy.

By employing a comparative case-study approach that covers a breadth of different NHS contexts and stakeholders, the study aims to produce generalisable knowledge about the process of sustainability, with practical and theoretical application across and beyond the health service. The overall clinical context of the four case-study sites--genetics--was chosen as being typical of other clinical areas that lack the political and popular interest of high-profile priority areas (*e.g*., cancer treatment or emergency department waiting times) and that cannot therefore rely on centrally driven change-management efforts. Instead, they require bottom-up agency through the work of frontline clinicians and managers, and while there may be particular lessons of interest to managers of clinical-genetics services, the findings will be relevant and generalisable to other areas of NHS provision that are similarly 'politically marginal' to the high-profile priorities and targets that drive much NHS behaviour [27]. The issues faced in sustaining new genetics services, then, are similar to those faced in other relatively marginal areas of NHS provision, and in an NHS faced with severe restraints on budget, the challenges facing such areas in achieving sustainability are likely to become more acute. The cross-sectoral nature of genetics provision makes it an especially suitable site for research of this kind, and the sampling strategy takes in case-study sites from primary, secondary, and tertiary care; sites with leaders from multiple professional groups; and sites in which locally developed and more centrally driven innovations are being sustained. Genetics is the common denominator across these sites, which are then sampled according to these key, theoretically informed variables of interest.

### Sampling, setting, and context

Four case-study sites from the earlier evaluation have been chosen as sites for this follow-up research. These have been sampled, following the theoretical sampling approach outlined by the likes of Eisenhardt and Yin [19,28], on the basis of consistencies and divergences in several characteristics that the literature, and our prior study, suggests are likely to be important in their paths to sustainability: clinical speciality, degree to which the original innovation derived from an evidence-based model, professional affiliation of service lead, sector in which organisational innovation is located, and mode by which initial postpilot sustainability was achieved. Of particular interest among these characteristics are the sector of the health service in which the innovation is being sustained (primary care versus secondary/tertiary hospital-based settings) [24] and the degree to which the innovation draws on some form of evidence base or is based on a locally designed approach to the reorganisation of care [16]. The former will have significant implications for how sustainability might be achieved (in terms of strategies and choice of funding), while the latter has particular implications for credibility of the organisational innovation with different groups of stakeholders. These variables are therefore given particular prominence in our sampling strategy. Table 1 gives details of the features of the four sites and how they embody the characteristics noted above.

**Table 1:** Characteristics of case-study sites

	Organisational innovation based on evidence-based model	Locally designed organisational innovation
Primary care-based organisational innovation	Case A Clinical speciality: cancer genetics Led by a nurse Commissioned by PCT	Case B General primary care genetics Led by a general practitioner Commissioned by PCT initially, funding currently halted

Hospital-based organisational innovation	Case C (tertiary care) Clinical speciality: cancer genetics Led by a consultant clinical geneticist Commissioned by a consortium of PCTs	Case D (secondary care) Other clinical speciality^a^ Jointly led by genetics and mainstream consultants Funded through integration into mainstream service

^a^To preserve anonymity, the clinical speciality of this site is not disclosed (since it was one of only a few). It is a lower-profile clinical area than cancer.

PCT = primary care trust.

Beyond these descriptive characteristics, the four cases differ in their subsequent paths into postpilot sustainability: while three have continued to enjoy ongoing funding, case B has since had funding from one source dropped and is seeking to replace this with alternative funding. Leads of all four sites, however, have agreed to involvement in the study, and the challenges faced by case B in reestablishing itself, having initially seemingly achieved sustainability, will further increase the richness provided by the sample.

### Data collection

The study will repeat those methods used in the prior evaluation, using in-depth interviews with key stakeholders, observations of relevant meetings, and documentary analysis. Interview schedules will be developed in the course of the review of the existing literature and secondary analysis of the prior evaluation's data set from these four sites; however, they are likely to cover a number of areas, the importance of which is already evident from our earlier work in these sites and others and knowledge of the literature. These areas include the changing nature of leadership in the sites; the development of the function and remit of the projects through time, especially during the transition from introducing the innovation through adapting it to the changing needs of the local health economy; the audiences whose input and/or approval is crucial to the sustainability of the projects; relationships with commissioners and other influential stakeholders, clinical and nonclinical; and the role of service-user involvement in determining need for projects and securing commitment from budget holders and decision makers.

Participants in the research will include those previously included plus a wider group of stakeholders with influence on medium-term sustainability (*e.g*., business managers, commissioners, primary care trust executives). Preliminary discussions with individuals at the four case-study sites suggest that the numbers of relevant stakeholders involved in the process vary from around 5 to 10, and so allowing for a degree of 'snowball sampling' through interviews, it is anticipated that around 25 to 45 interviews will be conducted. Observational work will include meetings relevant to the question of sustainability of the projects, and so the amount of observational work will depend on the number of such meetings taking place during the course of the study. Up to three meetings at each site will be observed to provide an understanding of current issues and how these are negotiated among the stakeholders involved in the projects. Interview schedules, observation methods, and documentary analysis will pay attention to areas considered important in sustainability from the earlier research and the literature (*e.g*., leadership, policy context, collaboration across boundaries, plus the specific areas noted above) but will remain open to issues that emerge through data collection.

### Data analysis

There will be two stages of data analysis. The first stage will involve a secondary analysis of data collected in the four sites in the course of the earlier evaluation. This will involve GPM (who was the lead researcher at the four case-study sites in the earlier evaluation) and the researcher, who will independently review transcripts from the original study and reanalyse them in terms of challenges and solutions around sustainability, establishment, and routinisation. This secondary analysis, along with review of the relevant literature, will help to inform interview schedules, observation, and documentary analysis during the fieldwork stage of the project.

Following the fieldwork, the newly collected data will be subjected to analysis led by the researcher but involving input from the whole team and combined with the findings from the secondary analysis of the data from the earlier evaluation. Given the limited time available in the context of a one-year project, a key issue in ensuring that this analysis is fit for our purposes will be balancing a focus on the issues known to be important from earlier work (the extant literature and our own work in this field) with an openness to unexpected findings that emerge from the data. Our approach to achieving this balance will involve using a model adapted from Ritchie and Spencer's [29] framework approach, which is especially well suited to policy-relevant research. This involves the mapping of the data onto predefined categories pertaining to the research question in a framework that enables both within-case analysis of how issues relate to one another (*e.g*., how 'sustainability strategy' relates to the sector in which the service is based) and cross-case analysis of these categories. Using this approach will also facilitate an explicitly longitudinal understanding of the data, with data categories subdivided according to the point in time at which data were collected, permitting a comparative analysis of how these issues have developed and become reframed through time. This approach will, however, be complemented by a more inductive mode of analysis, whereby GPM and the researcher will code data independently of one another at each site, identifying extra categories considered to be of importance to the research question, additional to those predefined on the basis of the literature and the reanalysis of data from the original evaluation. By combining the top-down framework approach with a certain amount of bottom-up (but focused) inductive analysis, the project will make the best use possible of the limited time available to ensure an analysis that takes into account existing knowledge, remains open to new findings in what is still a developing field, and, above all, is clearly focused on the research question.

## Competing interests

The authors declare that they have no competing interests.

## Authors' contributions

GPM conceived the idea for the study and led the intellectual development, funding application, and realisation. GC, RF, and RM contributed to the drafting and development of the study. All authors reviewed and agreed on the final manuscript.
